# Volume-outcome relationship and minimum volume regulations in the German hospital sector – evidence from nationwide administrative hospital data for the years 2005–2007

**DOI:** 10.1186/s13561-018-0204-8

**Published:** 2018-09-26

**Authors:** Corinna Hentschker, Roman Mennicken, Antonius Reifferscheid, Jürgen Wasem, Ansgar Wübker

**Affiliations:** 1 Essen, Germany; 2 FOM University of Applied Sciences, Essen Landschaftsverband Rheinland, Cologne, Germany; 3 University Duisburg-Essen, Essen, Germany; 4 RWI, RUB and Leibniz Science Campus Ruhr, Hohenzollernstraße 1-3, 45127 Essen, Germany

**Keywords:** Volume, Hospital quality, Mortality, Access to care, I12, I18

## Abstract

**Background:**

This paper analyses the volume-outcome relationship and the effects of minimum volume regulations in the German hospital sector.

**Methods:**

We use a full sample of administrative data from the unselected, complete German hospital population for the years 2005 to 2007. We apply regression methods to analyze the association between volume and hospital quality. We measure hospital quality with a binary variable, which indicates whether the patient has died in hospital. Using simulation techniques we examine the impact of the minimum volume regulations on the accessibility of hospital services.

**Results:**

We find a highly significant negative relationship between case volume and mortality for complex interventions at the pancreas and oesophagus as well as for knee replacement. For liver, kidney and stem cell transplantation as well as for CABG we could not find a strong association between volume and quality. Access to hospital care is only moderately affected by minimum volume regulations.

**Conclusion:**

The effectiveness of minimum volume regulations depends on the type of intervention. Depending on the type of intervention, quality gains can be expected at the cost of slightly decreased access to care.

## Background

Since the study of Luft et al. [1] the relationship between case volume and outcome-quality has been debated in the scientific literature. The international literature provides broad evidence for the volume-outcome relationship for various conditions in several disciplines - e.g. transplantation medicine, cardiology, orthopedics, neurosurgery, oncology, urology and neonatology (Halm et al. [2]; Gandjour et al. [3]; Chowdhury et al. [4]). The majority of these studies indicates that hospitals, which perform more (surgical) procedures, tend to have better outcomes than hospitals that perform fewer.

Due to these international findings, minimum volume regulations were implemented by German hospital policy in 2004. The idea behind these regulations is to exclude hospitals with bad outcomes caused from not performing certain procedures frequently enough. Currently, they are defined for seven conditions (since 2004: liver transplantation, kidney transplantation, complex oesophagus interventions, complex pancreas interventions and stem cell transplantation, followed by knee replacement (2006) and premature births (2010)).

However, for Germany credible empirical evidence on the volume-outcome relationship for these services is rare. Recently, Nimptsch et al. [5] assessed the association between minimum caseload requirements and in-hospital mortality in Germany. Using hospital discharge data from 2006 to 2013 and applying regression methods they found that adjusted in-hospital mortality in hospitals with a caseload above minimum volume threshold is significantly lower than in hospitals with a caseload below the threshold for four indications (esophageal surgery, pancreatic surgery, kidney transplantation and total knee replacement). For liver transplantation, no significant difference in adjusted mortality was found and for stem cell transplantation a positive association was found. Other existing studies focus on knee replacement and pancreatic surgery. Results indicate reduced wound infection rates with increasing case volumes for knee replacement (Geraedts et al. [6], Ohrmann et al. [7]). Recently, Krautz et al. [8] found, that patients who are undergoing major pancreatic resections have improved outcomes if they are admitted to higher volume hospitals. Other German studies focus on different conditions (Hentschker and Mennicken [9], Hentschker and Mennicken [10]) that are not included in the regulations.

Moreover, the potential impact of minimum volume regulations in Germany is scarce. Existing evidence suggest, that so far, in Germany minimum volume regulations have never been executed in the intended way (de Cruppé et al. [11], de Cruppé et al. [12], Peschke et al. [13], de Cruppé et al. [14], de Cruppé and Geraedts [15]). This can be partially explained by some exceptions from minimum volume regulations, for example to ensure access to hospital services. However, several hospitals treat patients in minimum volume conditions without achieving the minimum volume threshold and without fulfilling any legal exception. This shows that the regulation is not executed in the intended way. However, in the Hospital Structures Act in 2016 minimum volume regulations shall be drawn up in a legally secured manner.

This paper analyses the volume-outcome relationship and assesses the minimum volume regulations in the German hospital sector. It contributes to the literature in the following ways. First, it is one of the first studies (besides Nimptsch et al. [8]) which systematically evaluate the volume-outcome relationship for seven conditions that are affected by the German minimum volume regulations. This is important, as it is not clear whether results from other health systems are transferable to the German context and credible national empirical evidence is necessary. In comparison to Nimptsch et al. [8] we extend their assessment by not only comparing outcomes for hospitals below and above the minimum-volume thresholds, thus directly focusing on the general volume-outcome relationship for these indications. In this context, we analyze the relationship between volume and outcome with alternative econometric specifications (e.g. by different volume tertiles). Additionally, we simulate the consequences of withdrawing hospitals from performing a specific services for hospital access for different “hospital closure scenarios”. Moreover, we focus on an early period shortly after the introduction of minimum volume regulations. This focus might provide an assessment of the volume-outcome relationship to a quite unselective sample of the whole German hospital market, as hospitals did not adhere to the minimum volume regulation shortly after the introduction (see de Cruppé et al. [11] and Peschke et al.13]).

Second, despite international evidence generally supporting a positive volume outcome relationship (e.g. Halm et al. [2]), the magnitude of the associations differs widely across studies and the clinical and policy relevance of these findings is complicated by methodological limitations of many studies. For example, studies have shortcomings in controlling for differences in patient disease severity between high and low volume hospitals (e.g. Halm et al. [2], Gandjour et al. [3], Chowdhury et al. [4]). Our study uses comprehensive administrative data containing detailed information on patient health status. Third, our study uses a full sample of data from the unselected, complete German hospital population. This allows us to examine actual hospital case volumes. Existing studies are generally restricted to a specific group, e.g. Medicare patients (e.g. Barker, Rosenthal and Cram [16]). Fourth, by simulating whether minimum volume standards affect patient travel times, the study also sheds light on whether a trade-off exists between potential quality gains and reduced access to care for the regulated procedures. This potential trade-off is a major concern for German health policy. Withdrawing hospitals not meeting the minimum volume standards from performing the procedures has been studied by de Cruppé et al. [12] 2007, de Cruppé et al. [17], Geraedts et al. [18], Geraedts et al. [19] or Hentschker and Mennicken [9]. In comparison to these studies we do not only provide comparisons of travel times for the whole patient population in each condition, but also for the specific group of affected patients, i.e. patients with increasing travel times due to the closure of the nearest hospital in the specific service. This specific focus including only the people affected provides additional insights of the impact of minimum volume regulations on access to care.

## Methods

### Data and econometric model

The analysis is based on an administrative data set for the years 2005, 2006, and 2007.^1^ It is a sample of all inpatients in Germany (around 16.0 million (2005) to 16.6 million DRG-inpatients per year) - except psychiatric cases - treated in around 1700 hospitals. The data set includes detailed information on the patient; for example, age, gender, length of stay, diagnosis, procedure codes, patient admission date, and whether the patient died in the hospital (mortality as discharge reason). Moreover, structural information about each hospital is provided: ownership type, numbers of beds and teaching status.

This analysis focuses on six conditions which were governed by minimum volume regulations during the investigation period: liver transplantation (liver), kidney transplantation (kidney), oesophagus interventions (oesophagus), pancreas interventions (pancreas), stem cell transplantation (stem cell), and knee replacement (knee). Additionally, coronary artery bypass graft (CABG) surgery is also part of minimum volume regulations since its introduction, but minimum volume thresholds were never defined (G-BA [20], G-BA [21], G-BA [22]). For this reason CABG is also considered in this study to potentially derive policy implications for this condition. In the USA, the Leapfrog Group (2011) [23] recommends 450 CABG procedures per hospital. Such a minimum volume threshold seemed too high for the German actual medical care situation, with generally much smaller hospitals compared to the USA. For this reason, we choose a minimum volume threshold of 200.

Our analyzed conditions are identified with the procedure codes of the Federal Joint Committee (G-BA [20], G-BA [21], G-BA [22]). For CABG we use the definition of Mansky et al. [24]. Due to yearly updates of the minimum volume regulations of the Federal Joint Committee, procedure codes change slightly during the observation period. For example there are two additional procedure codes in 2007 for the definition of knee replacements. Therefore, the relevant conditions are identified by using the procedure codes of the respective year. We have to exclude patients with missing patient characteristics. Furthermore, we drop patients with discharge reason transfer (to another hospital) because we cannot determine the outcome of these patients.

We use in-hospital mortality as outcome measure in our analysis. Mortality is the most frequently used endpoint for volume-outcome analyses because it is the most severe clinical outcome (e.g. Cash et al. [25]; Fechner et al. [26]; Smith et al. [27]). Compared to other outcomes, mortality has the advantage of its robustness against hospitals’ individual coding behavior (AOK-Bundesverband et al. [28]). However, mortality is also a rare event – at least for certain conditions. This impedes the identification of statistically meaningful differences for hospitals with low case numbers. According to the literature, one possible approach is to eliminate hospitals with less than five expected death per year (Ash et al. [29]). However, this approach appears less rewarding in the context of the study due to its focus on hospitals with very low case volumes. To account for this, we choose three functional forms of case volume. First, we use the logarithm of case volume, which accounts for a decreasing effect of case volume on outcome with increasing volume. Second, we classify the case volume of hospitals in tertiles, which ensures a sufficient number of patients in every group (Hentschker and Mennicken [10]). In every tertile are approximately the same number of patients and we can distinguish between patients who are treated in low, medium, and high volume hospitals. Third, we specify a binary variable, which is 1 for patients who are treated in hospitals that achieve the minimum volume threshold and 0 otherwise. This variable should reflect whether the minimum volume thresholds have a significant impact on mortality.

To account for other factors which influence mortality besides case volume, we include several covariates in our empirical model. To reflect the impact of patient-specific factors on mortality risk, information on age, gender and especially the comorbidity of the patient must be considered. To account for number and severity of the comorbidities, we use the Charlson Comorbidity Index (CCI). The CCI considers 17 different comorbidities, each with a specific severity weight, which add to a total comorbidity score. A higher comorbidity score reflects a higher severity of illness, which is associated with an increased mortality risk (Charlson et al. [30]). Depending on their comorbidity score patients are divided into four risk-groups: CCI = 0, CCI = 1–2, CCI = 3–4, or CCI > = 5. Furthermore, it is controlled for different main diagnosis within one condition and the admission status (scheduled admission, emergency, transfer). Additionally, we include a binary variable for weekend or holiday admissions, because of a potentially higher mortality risk during those days (Cram et al. [31]).

Moreover, several hospital characteristics besides case volume are included in the model. Referring to Milcent [32], information about the ownership type is considered. Furthermore, university hospitals are represented by a binary indicator variable, because of tendencies to treat patients with more severe (co-)morbidities (Heyder [33]). To account for within-hospital correlation of mortality, standard errors are clustered at hospital level. Referring to Hentschker and Mennicken [8] we estimate the effect of volume on outcome with the following regression:

$$ {\mathrm{y}}_{\mathrm{ih}}={\upalpha}_0+{\mathrm{vol}}_{\mathrm{h}}{\upbeta}_1+\mathbf{x}{\hbox{'}}_{\mathrm{ih}}{\upbeta}_2+\mathbf{k}{\hbox{'}}_{\mathrm{h}}{\upbeta}_3+{\upvarepsilon}_{\mathrm{ih}} $$

**Table:** 

*y*_*ij*_	= mortality
*α*_0_	= constant
*vol*_*h*_	= case volume
*β*	= regression coefficients
*x*´_*ih*_	= vector of patient characteristics
*k*´_*h*_	= vector of hospital characteristics
*ε*_*ih*_	= error term
*i*	= patient index
*h*	= hospital index

This linear probability model is estimated by ordinary least squares. Our dependent variable *y*_*ih*_ is specified as a binary variable, 1 if patient died in hospital and 0 otherwise, for every patient *i* in hospital *h*. Case volume *vol* is specified depending on the functional form in the three different specifications. As mentioned above procedure codes change slightly during the observation period. Therefore we apply regressions for each year separately and do not exploit variation over time in our empirical specifications.^2^

### Accessibility analysis

In addition to the econometric analysis of the volume-outcome relationship, the impact of the minimum volume regulations on the accessibility of hospital services is examined [34]. Accessibility to hospital services is measured by travel times of patients to the according hospitals with different indicators. On the one hand we calculate actual travel times of patients to hospitals, i.e. travel of patient to the hospital they chose (“Status-quo-scenario”). On the other hand we calculate minimum travel times for different closing scenarios. In the closings scenarios we simulate that hospitals below the minimum volume thresholds are excluded from providing care (as described below). As we have the individual ZIP codes of all patients, we show changes in average travel times for all patients within a ZIP code area. We use over 8000 residential 5-digit ZIP code areas in Germany. To calculate travel times, we use the Stata command “traveltime”. We follow the approach of Hentschker and Mennicken (2015) [9]. As a first step, hospitals not achieving minimum volume thresholds are identified. The patients of these hospitals have to be redistributed to other hospitals which still provide the specific service. This implies longer travel times for the affected patients.

Sometimes patients do not choose the nearest hospital for treatment. This can lead to decreasing travel times in the simulation. Because we are interested in changes in access due to the minimum volume regulations, we assign minimum travel times to the patients, irrespective of whether the patient has been treated in the nearest hospital providing the respective procedure. For the following simulations, we exclude hospitals with a case volume below three cases. These hospitals are not relevant for care provision and should therefore not enter the simulation process. Additionally, we have to exclude patients with missing ZIP code, because we cannot assign travel times to hospitals for these patients.

Concerning the redistribution of patients, two different closure-scenarios are applied. The first scenario, “immediate closure”, models a simultaneous market exit of all hospitals not achieving the minimum volume threshold in the respective condition. The affected patients are allocated to the next nearest hospital from their place of residence which provides the same treatment. The second scenario, “successive closure”, models an iterative closing process. In each step the hospital with the smallest case volume is closed for the specific hospital service, and its patients are diverted to the next nearest hospital. This process is repeated until all hospitals achieve the minimum volume threshold for the specific condition. The main difference between the two scenarios is the opportunity for hospitals below the minimum volume threshold in the successive closure scenario to profit from the closure of the other hospitals with even lower case volumes and, hence, to increase case volume to the required threshold. We consider this scenario as the more realistic one.

Additionally to Hentschker and Mennicken (2015) [9], we do not only provide comparisons of travel times for the whole patient population in each condition, but also for the specific group of affected patients, i.e. patients with increasing travel times due to the closure of the nearest hospital in the specific service. This specific focus including only the people affected provides a more realistic insight of the impact of minimum volume regulations on access to care.

## Results

### Descriptive analysis

Table 1 summarizes number of patients and hospitals for each condition for every year. For most conditions the total number of patients increases from 2005 to 2007. Knee replacements are the largest subsample with over 120,000 patients treated in around 1000 hospitals each year, whereas liver transplantations are the condition with the smallest total case volume and the lowest number of hospitals. The amount of hospitals not achieving the minimum volumes varies by condition from 5% (kidney transplantation) to 75% (interventions at the oesophagus). Moreover, the changes of minimum volume thresholds in 2006 increased the share of hospitals not achieving minimum volume thresholds, but the number of hospitals providing the respective services stayed relatively constant. Although several hospitals fail to achieve minimum volumes, the vast majority of the patients are treated in hospitals achieving the required minimum volume threshold. Overall, the number of hospitals and the case volumes correspond with the data reported by other studies (Peschke et al. [14]; Geraedts et al. [24]; de Cruppé et al. [35]).

**Table 1:** Overview of number of patients and hospitals for all conditions from 2005 to 2007

Condition	Year	Number of patients	Number of hospitals	Average case volume	Minimum volume threshold	Hospitals achieving minimum volume threshold (%)	Patients treated in these hospitals (%)
Liver transplantation	2005	941	22	42.8	10	81.8	96.2
	2006	1005	22	45.7	20	68.2	89.8
	2007	1118	22	50.8	20	77.3	94.5
Kidney transplantation	2005	2627	42	62.5	20	92.9	97.9
	2006	2728	42	65.0	25	90.5	97.8
	2007	2902	42	69.1	25	95.2	98.6
Complex interventions at the oesophagus	2005	3063	436	7.0	5	36.2	79.5
	2006	3249	411	7.9	10	25.1	63.1
	2007	3361	437	7.7	10	24.0	64.5
Complex interventions at the pancreas	2005	7795	708	11.0	5	47.0	88.5
	2006	8330	712	11.7	10	32.2	77.9
	2007	9152	691	13.2	10	40.1	82.3
Stem cell transplantation	2005	5522	102	54.1	12	70.6	97.5
	2006	5940	94	63.2	25	61.7	91.5
	2007	5744	101	56.9	25	60.4	92.5
Knee replacement	2005	118,269	1055	112.1	–	–	–
	2006	124,693	1017	122.6	50	78.2	96.0
	2007	134,782	1004	134.2	50	83.8	97.2
CABG	2005	43,501	95	457.9	(200)^a^	77.9	99.1
	2006	39,254	102	384.8	(200)^a^	69.6	97.8
	2007	38,569	101	381.9	(200)^a^	69.3	96.5

Note: ^a^ No official minimum volume threshold exists; a hypothetical minimum volume threshold of 200 is assumed

Table 2 shows descriptive statistics of patient and hospital characteristics in 2007 and comprises only patients which are also included in the regressions, i.e. patients with missing patient characteristics and discharge reason transfer are excluded. The diagnosis specific main diagnoses are shown in the Appendix in Table 5. In-hospital mortality varies by condition from 0.1% (knee replacements) to 17.7% (liver transplantations). On the one hand, the low mortality rates of knee replacements and CABG impede analysis of volume-outcome relations. On the other hand, the high case volumes in these conditions are advantageous from a statistical point of view. Patients receiving liver, kidney or stem cell transplantations are on average 50 years old. For all other conditions the average age is above 60 years. Besides knee replacement, male patients are more prevalent in all other conditions. In general, admission on weekend/holiday is more likely for conditions with a higher share of emergency cases. Again, knee replacement is an exception with the lowest emergency rate and yet still 17.5% weekend/holiday admissions. Moreover, the conditions with the highest mortality rates (liver, pancreas, oesophagus) also have the highest comorbidity score with a quarter of patients having a CCI-score above five. The university status of the hospitals is important for liver and kidney transplantations with the vast majority of patients being treated at university hospitals. One third of stem cell transplantations and CABG are performed in university hospitals. As university hospitals mostly have a public owner, the percentage of public hospitals is very high for these conditions.

**Table 2:** Descriptive statistics of patient and hospital characteristics (2007)

	Liver	Kidney	Oesophagus	Pancreas	Stem cell	Knee	CABG
Patient level							
Number of patients	1064	2885	3190	8854	5687	132,195	27,644
Mortality rate (%)	18.6%	1.8%	11.8%	10.1%	5.9%	0.1%	3.2%
Age (mean)	48.1	49.7	62.8	62.1	48.4	69.7	66.4
Male (%)	63.3%	62.3%	75.9%	57.5%	62.4%	32.3%	78.3%
Admission reason (%)							
Scheduled	32.6%	34.6%	80.9%	68.9%	80.2%	95.2%	63.2%
Emergency	52.7%	60.3%	13.6%	23.3%	14.1%	4.5%	9.3%
Transfer	14.7%	5.0%	5.5%	7.8%	5.7%	0.3%	27.5%
Weekend/holiday admission (%)	22.4%	24.1%	9.3%	12.1%	5.0%	17.5%	7.7%
Charlson comorbidity index (%)							
0	8.6%	18.7%	17.9%	27.1%	47.0%	65.2%	31.7%
1–2	22.7%	47.9%	35.0%	34.3%	28.4%	30.4%	46.8%
3–4	40.0%	26.3%	20.5%	16.2%	9.4%	3.7%	16.4%
> =5	28.7%	7.1%	26.6%	22.5%	15.2%	0.6%	5.1%
Hospital level							
Number of hospitals	22	42	415	680	100	999	98
Case volume (mean)	50.8	69.1	8.0	13.4	57.4	134.2	393.5
Ownership (%)							
Public	100.0%	95.2%	46.3%	45.0%	68.0%	42.2%	55.1%
Private non-profit	0.0%	2.4%	41.4%	42.2%	16.0%	40.0%	18.4%
Private for-profit	0.0%	2.4%	12.3%	12.8%	16.0%	17.7%	26.5%
University hospital (%)	95.5%	78.6%	9.2%	5.4%	35.0%	3.6%	34.7%

### Results of the econometric model

Table 3 shows the estimation results for each condition for every year. We find different results for the conditions. We find a highly significant negative relationship between case volume and mortality for complex interventions at the pancreas and oesophagus as well as for knee replacement supporting the volume-outcome relationship. For example, for complex pancreas interventions we find the following results. The left column shows results of the log specification for case volume. The coefficient of − 0.028 (year 2007) indicates that an increase of 1% in case volume reduces the probability of death by 0.028 percentage points. More precisely: a patient who is treated in a hospital with 10 cases has a probability of death of 12.8% (not shown in the table). An increase of 10 cases reduces the probability of death by 1.9 pp. to 10.9%. For the calculation of the changes in the probability of death, we take the “average” patient and set all variables of the model except case volume at their means. The middle columns display that for example in the year 2007 hospitals in the middle tertile (highest tertile) of case volumes have a 3.87 percentage points (5.03 percentage points) lower mortality rate than the hospitals in the lowest tertile. The right column shows that hospitals above the minimum-volume thresholds have a 5.97 percentage points lower mortality rate than hospitals below the minimum-volume threshold. These numbers relate again to complex pancreas interventions for the year 2007.

**Table 3:** Results of the econometric models

Condition	Year	OLS with logarithm of case volume		OLS with case volume tertiles (reference group: low case volume)				OLS with binary variable whether minimum volume threshold is achieved		Number of	
				Medium case volume		High case volume					
		Coeff.	S.E.	Coeff.	S.E.	Coeff.	S.E.	Coeff.	S.E.	Hospitals	Patients
Liver	2005	−0.0324*	(0.0175)	−0.0118	(0.0518)	− 0.0513	(0.0354)	−0.0531	(0.0588)	22	906
	2006	−0.0331	(0.0267)	−0.0027	(0.0356)	−0.0441	(0.0513)	−0.0383	(0.0448)	22	965
	2007	− 0.0414	(0.0255)	− 0.0263	(0.0520)	− 0.0279	(0.0381)	− 0.1285*	(0.0732)	22	1064
Kidney	2005	−0.0026	(0.0067)	0.0017	(0.0057)	−0.0010	(0.0066)	−0.0035	(0.0129)	42	2610
	2006	0.0001	(0.0055)	−0.0043	(0.0062)	0.0050	(0.0059)	−0.0013	(0.0128)	42	2699
	2007	−0.0102	(0.0064)	−0.0070	(0.0074)	−0.0065	(0.0072)	−0.0539***	(0.0156)	42	2885
Oesophagus	2005	−0.0292***	(0.0081)	−0.0098	(0.0166)	−0.0445*	(0.0250)	−0.0476***	(0.0177)	428	2898
	2006	−0.0306***	(0.0074)	−0.0284*	(0.0166)	−0.0668***	(0.0203)	−0.0422***	(0.0157)	405	3107
	2007	−0.0267***	(0.0075)	−0.0159	(0.0157)	−0.0262	(0.0203)	−0.0186	(0.0147)	415	3190
Pancreas	2005	−0.0268***	(0.0050)	−0.0494***	(0.0103)	−0.0776***	(0.0135)	−0.0586***	(0.0133)	696	7480
	2006	−0.0280***	(0.0053)	−0.0372***	(0.0105)	−0.0568***	(0.0161)	−0.0632***	(0.0112)	702	8031
	2007	−0.0280***	(0.0051)	−0.0387***	(0.0095)	−0.0503***	(0.0150)	−0.0597***	(0.0112)	680	8854
Stem cell	2005	0.0033	(0.0053)	0.0123	(0.0140)	0.0044	(0.0109)	−0.0011	(0.0184)	100	5489
	2006	0.0042	(0.0085)	0.0383**	(0.0166)	0.0043	(0.0164)	0.0139	(0.0151)	94	5883
	2007	0.0056	(0.0059)	0.0290**	(0.0130)	0.0126	(0.0139)	0.0258*	(0.0134)	100	5687
Knee	2005	−0.0005***	(0.0002)	−0.0002	(0.0003)	−0.0006*	(0.0003)			1047	115,401
	2006	−0.0007***	(0.0002)	−0.0009***	(0.0003)	−0.0012***	(0.0003)	−0.0026***	(0.0010)	1008	122,150
	2007	−0.0004***	(0.0002)	−0.0003	(0.0003)	−0.0006**	(0.0002)	−0.0005	(0.0008)	999	132,195
CABG	2005	−0.0011	(0.0046)	−0.0008	(0.0086)	−0.0064	(0.0078)	0.0012	(0.0105)	94	30,633
	2006	−0.0009	(0.0050)	0.0004	(0.0070)	−0.0037	(0.0081)	−0.0021	(0.0099)	101	27,891
	2007	−0.0103***	(0.0037)	−0.0052	(0.0055)	−0.0107*	(0.0061)	−0.0157*	(0.0094)	98	27,644

Note: The table shows the effect of case volume on mortality for different specifications of case volume. All regressions are estimated with the following covariates: age, male, charlson comorbidity index (1–2, 3–4, > = 5), admission reason (emergency, transfer), weekend/holiday admission, diagnosis specific main diagnoses, ownership (private not-for-profit, private for-profit), and university hospital. The tertiles divide the sample in three parts based on the case volume of hospitals. Hence, it is possible to distinguish patients treated in low, medium and high volume hospitals

*Significant at 10%, **Significant at 5%, ***Significant at 1%

In sum, the effect of case volume on mortality for pancreas interventions is of substantial size. The effects are of similar magnitude for complex interventions at oesophagus. It is much lower and close to zero for knee replacements, because of the low overall mortality rate in this condition.

In contrast, for liver and kidney transplantation as well as for CABG only few statistically significant negative coefficients between the case volume and mortality are identified which cannot support a volume-outcome relationship. Also for stem cell transplantation we could not find any evidence of a relationship between volume and outcome.

### Results of the accessibility analysis

The observed travel times and the minimum travel times for status quo and both closing scenarios are presented in Table 4. The travel times were calculated for the whole patient population in each condition as well as only for the patients affected, i.e. patients with increasing travel times due to the closure of the hospital in the specific service. Table 4 reads as follows: The four column on the right hand side of Table 4 provide information for the whole patients. For example, actual travel time for Liver patients was 69 min. Minimum travel times for Liver patients are on average 45 min in status quo with a maximum of 166 min to the nearest hospital. Ninety-five percent of all patients in our sample would reach a hospital within 98 min. In this baseline scenario, all 22 hospital still provide services. In scenario 1 “immediate closure”, the five hospitals of the first quintile lose its authorization to treat Liver patients leaving 17 hospitals in the sample. This scenario leads to an increase in average travel time by more than 4 min. The maximum travel time in this scenario would be 167 min with a 95% percentile of 106 min. In comparison with scenario 1, a stepwise introduction (scenario “successive closure”) has a similar impact on travel times. Average travel times are around 48 min with a maximum time of 167 min. Ninety-five percent of the patients reach the nearest hospital within 106 min. The four columns on the left hand side of Table 4 present the according information for the patients that are really affected by the closure. It becomes obvious that for the affected patients travel time increases strongly by hospital closure. E.g. for affected people minimum average travel time increases sharply from 36 min in the status quo to 70 min in the immediate closure scenario and to 68 min in the successive closure scenario.

**Table 4:** Results of the accessibility analysis for 2007

	All patients				Affected patients			
	Observed travel time	Minimum travel time			Observed travel time	Minimum travel time		
		Status quo	Immediate closure	Successive closure		Status quo	Immediate closure	Successive closure
Liver								
Average	69.0	45.0	49.3	48.4	43.7	36.2	70.0	67.8
Standard deviation	67.9	28.1	30.4	30.2	42.7	25.6	34.4	39.6
Minimum	2	2	2	2	4	4	13	13
Maximum	497	166	167	167	276	108	167	167
25% percentile	26	23	25	25	17	16	48	35
50% percentile	49	41	45	43	32.5	30.5	63.5	56
75% percentile	89	60	67	65	57	48	88	93
95% percentile	193	98	106	106	106	85	126	128
Min volume threshold	9	9	21	21				
Number of hospitals	22	22	17	18				
Number of patients	1041	1041	1041	1041	62	62	62	49
Kidney								
Average	52.4	38.2	38.9	38.9	36.8	32.6	50.7	50.7
SD	41.6	23.5	23.8	23.8	18.8	15.5	15.5	15.5
Minimum	2	2	2	2	5	5	23	23
Maximum	451	130	130	130	83	61	82	82
25% percentile	23	19	20	20	25	25	41	41
50% percentile	43	33	34	34	38	34	53	53
75% percentile	71	54	55	55	48	45	61	61
95% percentile	125	84	84	84	61	55	72	72
Min volume threshold	19	19	26	26				
Number of hospitals	42	42	40	40				
Number of patients	2835	2835	2835	2835	41	41	41	41
Oesophagus								
Average	35.7	19.6	26.2	23.5	24.6	16.0	29.5	26.1
SD	44.9	13.9	17.6	16.1	36.4	11.9	18.0	16.6
Minimum	0	0	0	0	0	0	2	0
Maximum	495	85	107	104	395	64	100	85
25% percentile	12	9	12	11	9	7	15	13
50% percentile	22.5	16	22	20	16	13	25	22
75% percentile	43	27	37	34	27	21	41	36
95% percentile	102	47	60	56	69	39	63	59
Min volume threshold	3	3	11	10				
Number of hospitals	270	270	117	150				
Number of patients	3080	3080	3080	3080	842	842	842	598
Pancreas								
Average	33.2	15.6	19.1	18.1	17.4	12.8	24.4	22.9
SD	43.2	10.6	13.0	12.5	21.8	9.0	14.5	14.1
Minimum	0	0	0	0	0	0	1	1
Maximum	535	84	89	87	324	58	85	67
25% percentile	11	7	9	9	7	6	12	12
50% percentile	19	13	16	15	13	11	21	20
75% percentile	38	21	27	25	21	17	34	31
95% percentile	104	36	44	43	43	30	52	51
Min volume threshold	3	3	10	10				
Number of hospitals	502	502	303	338				
Number of patients	8733	8733	8733	8733	1111	1111	1111	829
Stem cell								
Average	51.4	30.7	33.2	32.5	37.2	25.3	39.3	37.1
SD	49.6	19.8	20.7	20.5	39.6	16.3	19.4	19.8
Minimum	0	0	0	0	2	1	4	4
Maximum	481	117	117	117	251	109	109	109
25% percentile	20	15	16	16	16	14	23	21
50% percentile	39	26	29	28	26	22	37	34
75% percentile	65	42	46	45	44	32	52	50
95% percentile	138	70	73	72	113	58	73	72
Min volume threshold	4	4	25	25				
Number of hospitals	90	90	65	68				
Number of patients	5517	5517	5517	5517	317	317	317	264
Knee								
Average	26.7	12.9	13.6	13.5	22.3	11.8	16.8	16.5
SD	32.1	7.8	8.2	8.1	30.2	7.3	9.2	8.9
Minimum	0	0	0	0	0	0	0	0
Maximum	553	92	92	92	465	46	62	53
25% percentile	11	7	7	7	9	6	10	10
50% percentile	19	11	12	12	15	10	16	15
75% percentile	31	18	18	18	25	16	22	22
95% percentile	68	27	29	29	60	26	34	34
Min volume threshold	3	3	50	50				
Number of hospitals	974	974	845	853				
Number of patients	133,389	133,389	133,389	133,389	3290	3290	3290	2924
CABG								
Average	45.1	31.3	32.5	32.5	45.2	29.7	49.5	48.6
SD	38.8	18.5	19.3	19.2	46.9	19.2	25.4	24.7
Minimum	0	0	0	0	0	0	2	2
Maximum	497	113	113	113	458	96	110	106
25% percentile	19	17	17	17	16	15	29	27
50% percentile	34	28	28	28	29	25	48	49
75% percentile	59	43	45	45	62	41	65	65
95% percentile	113	66	69	68	132	68	96	92
Min volume threshold	3	3	200	200				
Number of hospitals	83	83	71	72				
Number of patients	37,965	37,965	37,965	37,965	1133	1133	1133	943

**Fig. 1:**
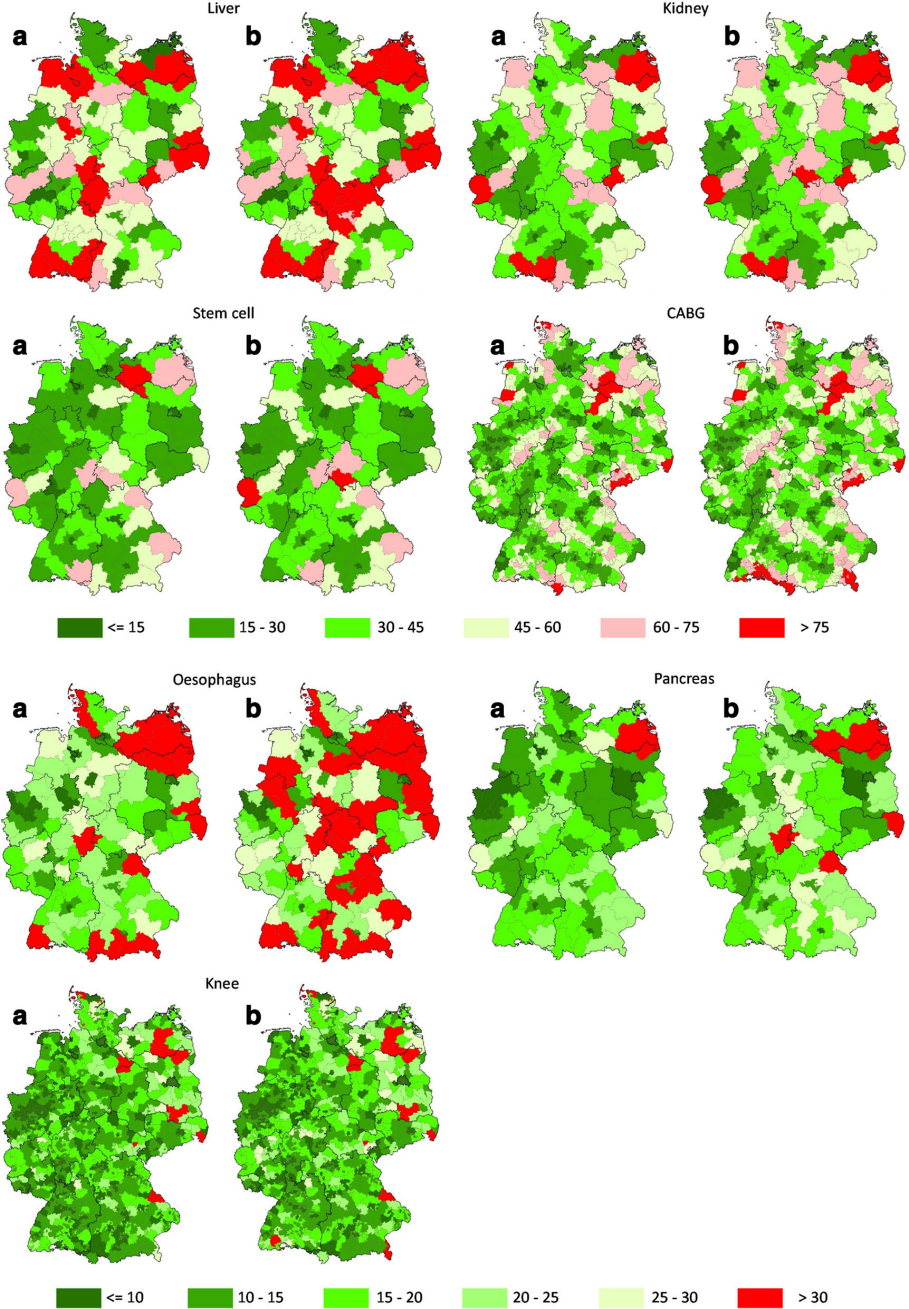
Minimum travel times in minutes in Status quo (**a**) and after stepwise introduction of the minimum volume threshold (**b**), 2007

Generally, the impact of hospital closures for liver, kidney and stem cell transplantations are rather small in the whole population. Median minimum travel times increase only by 2 min maximum when comparing status quo and successive closure scenario. If only affected patients are considered, the closure of even a small number of hospitals leads to a strong increase in travel times. However, it is observable that in some regions already in the status quo patients need more than 75 min to the nearest hospital (see Fig. 1) and hence, after the hospital closures only slight deteriorations in access are noticeable. Moreover, the access to hospital services is graphically depicted to show differences in access in different regions.

In contrast to the transplantations, travel times are much lower for interventions at the oesophagus and pancreas. The closure of hospital below the minimum volume threshold leads to an increase in median travel times of 2 to 4 min. This is quite a small increase considering the fact that a substantial part of hospitals do not achieve the minimum volume threshold. However, closing affects regions differently; in particular, for interventions at the oesophagus, access deteriorates enormously in many regions in Germany (see Fig. 1), i.e. in many regions patients need more than 30 min to reach the nearest hospital after stepwise introduction of the minimum volume threshold.

More than 950 hospitals provide knee replacements. The closure of hospitals not achieving the minimum volume regulations threshold does not lead to any deterioration in access. For directly affected patients, travel time increases by 4 min. We again see some regional variation in access to this treatment. In some regions patients need longer than 30 min to reach a hospital, but this is not a result of the simulation; it is already the situation in the status quo.

CABG has a high case volume with a comparably low number of hospitals treating this condition. Considering all patients, no increase in median travel times is observable after closure of hospitals below the minimum volume thresholds. However, affected patients have an increase in median travel time of 24 min which is comparable to the increase in travel times by liver transplantations. Even before the simulation, in some regions patients need more than 60 min to reach a CABG hospital. Access has deteriorated after the simulations in some regions.

In summary, the impact of hospital closures on travel time is generally higher i) the lower the number of existing hospitals is and ii) the higher the number of closures is. It makes a substantial difference whether all patients are considered or whether only affected patients are analyzed. Particularly for the latter, we see a strong impact from hospital closures. Finally, regions are affected differently by closings.

## Discussion/limitations

This study has two major strengths: First, the study conducts a comprehensive analysis for seven conditions, which includes the investigation of volume-outcome relationship as well as service accessibility. Second, the data set represents a complete sample of all German inpatients for three consecutive years including detailed information on patient health status. Moreover, although mortality is the most common quality outcome, some volume-outcome studies include further quality measures. However, no reliable information was available regarding other quality outcomes (e.g. complications). Further studies should take other outcome variables such as complication rates or other quality indicators into account. With regard to risk the control variables (age, sex and comorbidities, etc.) should cover central patient-related risk factors. For particular conditions, additional clinical data could be useful. For example the “Model for End-stage Liver Disease” (MELD)-Score, that represents the degree of severity of a liver disease (Wiesner et al. [36]), could improve risk-adjustment for the condition “liver transplantation”. Regarding the controls on the hospital level, further structural factors (e.g. technical equipment/infrastructure) may be appropriate, but were not available.

Another limitation is that our results show only a correlation between case volume and mortality. Further research might exploit the question of causality more deeply. One approach would be an instrumental variable strategy as done by Hentschker and Mennicken [10] or Seider et al. [37]. The volume-outcome relationship can be explained by two hypotheses with reverse causality directions. The practice-makes-perfect hypothesis assumes that a high case volume leads to better outcomes due to learning effects and with this the improvement of skills. In contrast, the selective-referral hypothesis states that a good outcome leads to higher case volumes. The idea behind this hypothesis is the assumption that, for example, primary physicians know where the quality hospitals are. Another bias can occur due to unobserved patient heterogeneity (omitted variable bias). If we are not able to control for all patient characteristics which are correlated with volume and the outcome variable our results will be biased [10]. An instrumental variable approach might correct for reverse causality and omitted variable bias, but we did not find valid instruments. Moreover, the analysis relies on 10 year old data. Due to data access limitations, we were not able not run an analysis with current data. However, even if meanwhile some changes in the hospital sector occurred, we are confident that the underlying relationships driving the results are still existent. Moreover, as we focus on a time period at an early period of minimum volume regulation in Germany and studies indicate (e.g. that at least at that time) this regulation was not effective in Germany. Thus, focusing on this period has the advantage that we are able to assess the volume-outcome relationship on a quite unselected (i.e. not by minimum thresholds affected) hospital population. Finally, our analysis relies on in-hospital mortality and just reflects the time within the hospital. Future studies might additionally consider outpatient mortality and other outcomes like complications.

## Conclusion

This study constitutes a comprehensive analysis of minimum volume regulations in Germany. Based on a full sample of all inpatients from 2005 to 2007, volume-outcome relationships are investigated for seven conditions. This study partially confirms international evidence on volume-outcome relationships. In particular, significant negative associations between case volume and in-hospital mortality are identified for oesophagus interventions, pancreas interventions, and knee replacements. For the other conditions, no clear volume-outcome relationship could be identified. This confirms generally results from Nimptsch et al. [7] who focus directly on the minimum volume thresholds and find also a significant negative relationship for kidney transplantations.

Moreover, we found that a relevant share of hospitals did not achieve minimum volume thresholds in each year but still provided these services. Thus, in the study period the introduction of minimum volume regulations seemed to have a limited impact on the supply side. The amount of hospitals not achieving the minimum volume thresholds varies by condition from 5% to 75%. Also, the modification of the thresholds in 2006 did not show relevant effects. These results correspond with other investigations [10, 15]. However, our findings demonstrate the potential steering effect minimum volumes could have if minimum volumes would be strictly implemented in Germany. Moreover, the accessibility analysis shows that a strict implementation of the minimum volume regulations could also result in a reduced accessibility of hospital in certain regions, particularly for oesophagus interventions in Eastern Germany. In general, patients show a high mobility, as the observed travel times are noticeably higher than the minimum travel times.

Recent legislative changes in Germany prohibiting compensation of services in hospitals that do not reach the minimum volume threshold will probably increase the proportion of hospitals which are compliant with the minimum volume regulations. Based on our findings, these regulations might induce quality gains at the cost of moderately decreased access to these services.

In comparison to other countries the German minimum volume standards appear relatively moderate. For example the threshold in the Netherlands for interventions at the oesophagus and pancreas is 20 [38, 39]. In France there is even a threshold of 30 for pancreatic resections [39]. We observed significant negative associations between case volume and in-hospital mortality for these indications. Consequently, an adjustment of these standards should be discussed.

## Appendix

**Table 5:** Main diagnoses of the conditions

Main diagnosis	Share
Liver transplantation	
Diseases of liver	55.8%
Malignant neoplasms of liver	16.9%
Liver donor	4.4%
Other main diagnosis	22.8%
Kidney transplantation	
Chronic kidney disease	93.2%
Other main diagnosis	6.8%
Complex interventions at the oesophagus	
Malignant neoplasms of oesophagus or stomach	88.8%
Other diseases of oesophagus or gastric ulcer	5.6%
Other main diagnosis	5.5%
Complex interventions at the pancreas	
Malignant neoplasms of pancreas	50.5%
Other disease of pancreas	22.6%
Malignant neoplasm of stomach, small intestine, or colon	8.3%
Other main diagnosis	18.6%
Stem cell transplantation	
Multiple myeloma and malignant plasma cell neoplasms, myeloid or lymphoid leukaemia	60.7%
Lymphoma	20.5%
Malignant neoplasms of testis	4.5%
Other main diagnosis	14.2%

Note: No covariates for knee replacement included, 98% of the patients have the main diagnosis arthrosis of knee. No covariates for CABG included, 98% of the patients have the main diagnosis chronic ischaemic heart disease or angina pectoris

## Abbreviations

Coeff: Coefficient
OLS: Ordinary Least Squares
S.E.: Standard Error

## Footnotes

1 The administrative data of §21 KHEntgG was used as part of a cooperation agreement for the further development of the DRG-system from April, 1st 2011 between the RWI and the BKK Federal Association.

2 For example, the numbers of knee replacement for 2006 and 2007 are not comparable due to a change in the definition of procedure codes by G-BA. In 2007 two additional procedure codes (5–822.a and 5–822.b) were used for the definition of knee replacement which could yield to an increase in the total number of knee replacements. We do not know exactly whether and to what extent these new procedure codes are responsible for the increase. As a comparison of total case volume between the years might be misleading, we abstain from exploiting longitudinal data in our analysis.

## References

[1] Luft HS, Bunker JP, Enthoven AC (1979). Should operations be regionalized? The empirical relation between surgical volume and mortality. New Engl J Med.

[2] Halm EA, Lee C, Chassin MR (2002). Is volume related to outcome in health care? A systematic review and methodologic critique of the literature. Ann Intern Med.

[3] Gandjour A, Bannenberg A, Lauterbach KW (2003). Threshold volumes associated with higher survival in health care – A systematic review. Med Care.

[4] Chowdhury MM, Dagash H, Pierro A (2007). A systematic review of the impact of volume of surgery and specialization on patient outcome. Brit J Surg.

[5] Nimptsch U., Peschke D., Mansky T. (2016). Mindestmengen und Krankenhaussterblichkeit – Beobachtungsstudie mit deutschlandweiten Krankenhausabrechnungsdaten von 2006 bis 2013. Das Gesundheitswesen.

[6] Geraedts M, de Cruppé W, Blum K, Ohmann C (2008). Umsetzung und Auswirkungen der Mindestmengen. Deutsches Ärzteblatt.

[7] Ohmann C, Verde PE, Blum K, Fischer B, de Cruppé W, Geraedts M (2010). Two short-term outcomes after instituting a national regulation regarding minimum procedural volumes for total knee replacement. J Bone Joint Surg Am.

[8] Krautz Christian, Nimptsch Ulrike, Weber Georg F., Mansky Thomas, Grützmann Robert (2018). Effect of Hospital Volume on In-hospital Morbidity and Mortality Following Pancreatic Surgery in Germany. Annals of Surgery.

[9] Hentschker C, Mennicken R (2015). The volume-outcome relationship and minimum volume standards – empirical evidence for Germany. Health Econ.

[10] Hentschker Corinna, Mennicken Roman (2017). The Volume-Outcome Relationship Revisited: Practice Indeed Makes Perfect. Health Services Research.

[11] de Cruppé W, Malik M, Geraedts M (2014). Achieving minimum caseload requirements: an analysis of hospital quality control reports from 2004-2010. [Umsetzung der Mindestmengenvorgaben – analyse der Krankenhausqualitätsberichte Eine retrospektive Studie der Jahre 2004–2010]. Dtsch Arztebl Int.

[12] de Cruppé W, Malik M, Geraedts M (2015). Minimum volume standards in German hospitals: do they get along with procedure centralization? A retrospective longitudinal data analysis. BMC Health Serv Res.

[13] Peschke D, Nimptsch U, Mansky T (2014). Achieving minimum caseload requirements: an analysis of hospital discharge data from 2005–2011. Dtsch Arztebl Int.

[14] De Cruppé W, Ohmann C, Blum K, Geraedts M. Evaluating compulsory minimum volume standards in Germany: how many hospitals were compliant in 2004? BMC Health Serv Res. 2007;7(165). 10.1186/1472-6963-7-165 PMC2204003 17941973

[15] de Cruppé W, Geraedts M (2016). Wie konstant halten Krankenhäuser die Mindestmengenvorgaben ein? Eine retrospektive, längsschnittliche Datenanalyse der Jahre 2006, 2008 und 2010. [How Steady are Hospitals in Complying with Minimum Volume Standards? A Retrospective Longitudinal Data Analysis of the Years 2006, 2008, and 2010.]. Zentralbl Chir.

[16] Barker D, Rosenthal G, Cram P (2011). Simultaneous relationships between procedure volume and mortality: do they bias studies of mortality at specialty hospitals?. Health Econ.

[17] de Cruppé W, Ohmann C, Blum K, Geraedts M (2008). Auswirkung der Mindestmengenvereinbarung auf die stationäre Versorgungsstruktur. [Influence of minimum volumes on the structure of inpatient care.]. Gesundheitswesen..

[18] Geraedts M, de Cruppé W, Blum K, Ohmann C (2010). Distanzen zu Krankenhäusern mit Mindestmengen-relevanten Eingriffen 2004 bis 2006. [Distances to Hospitals Performing Minimum Volume Relevant Procedures in Germany 2004 to 2006.]. Das Gesundheitswesen.

[19] Geraedts M, Kühnen C, de Cruppé W, Blum K, Ohmann C (2008). Unterschreitungen der Mindestmengen 2004: Begründungen und Konsequenzen. [Hospitals Failing Minimum Volumes in 2004: Reasons and Consequences.]. Das Gesundheitswesen.

[20] G-BA, Gemeinsamer Bundesausschuss: Vereinbarung des Gemeinsamen Bundesausschusses gem. §137 Abs. 1 Satz 3 SGB V für nach § 108 SGB V zugelassene Krankenhäuser (Mindestmengenvereinbarung) in Kraft getreten am 01. Januar 2005 (2005).

[21] G-BA, Gemeinsamer Bundesausschuss: Vereinbarung des Gemeinsamen Bundesausschusses gem. §137 Abs. 1 Satz 3 SGB V für nach § 108 SGB V zugelassene Krankenhäuser (Mindestmengenvereinbarung) in Kraft getreten am 21. März 2006 (2006).

[22] G-BA, Gemeinsamer Bundesausschuss: Vereinbarung des Gemeinsamen Bundesausschusses gem. §137 Abs. 1 Satz 3 SGB V für nach § 108 SGB V zugelassene Krankenhäuser (Mindestmengenvereinbarung) in Kraft getreten am 01. Januar 2007 (2007).

[23] Leapfrog Group (2011). Factsheet – evidence-based hospital referral, revision 03/21/11.

[24] Mansky T, Niptsch U, Winklmair C, Vogel C, Hellerhoff F (2011). G-IQI – German inpatient quality indicators (version 3.1).

[25] Cash H, Slowinski T, Buechler A, Grimm A, Friedersdorff F, Schmidt D, Miller K, Giessing M, Fuller TF (2012). Impact of surgeon experience on complication rates and functional outcomes of 484 deceased donor renal transplants: a single-Centre retrospective study. BJU Int.

[26] Fechner G, Seifert I, Hauser S, Müller SC (2012). Impact of a learning curve model in kidney transplantation on functional outcome and surgical complications in a small volume Centre: does size really matter?. Int Urol Nephrol.

[27] Smith BR, Hinojosa MW, Reavis KM, Nguyen NT (2008). Outcomes of esophagostomy according to surgeon’s training: general vs. thoracic. J Gastrointest Surg.

[28] AOK-Bundesverband, Forschungs- und Entwicklungsinstitut für das Sozial- und Gesundheitswesen Sachsen-Anhalt (FEISA), HELIOS Kliniken and Wissenschaftliches Institut der AOK (WIdO). Qualitätssicherung der stationären Versorgung mit Routinedaten (QSR), Abschlussbericht, Bonn. 2007.

[29] Ash AS, Posner MA, Speckman J, Franco S, Yacht AC, Bramwell L (2003). Using claims data to examine mortality trend following hospitalization for heart attack in medicare. Health Serv Res.

[30] Charlson ME, Pompei P, Ales KL, MacKenzie CR (1987). A new method of classifying prognostic comorbidity in longitudinal studies: development and validation. J Chronic Dis.

[31] Cram P, Hillis SI, Barnett M, Rosenthal GE (2004). Effects of weekend admission and hospital teaching status on in-hospital mortality. Am J Med.

[32] Milcent C (2005). Hospital ownership, reimbursement systems and mortality rates. Health Econ.

[33] Heyder R, Klauber J, Geraedts M, Friedrich J, Wasem J (2015). Die Bedeutung der Universitätskliniken in der regionalen und überregionalen Versorgung. Krankenhaus-Report 2015: Strukturwandel.

[34] Geraedts M, de Cruppé W, Blum K, Ohmann C (2010). Distanzen zu Krankenhäusern mit mindestmengen-relevanten Eingriffen 2004 bis 2006. Gesundheitswesen..

[35] de Cruppé W., Ohmann C., Blum K., Geraedts M. (2008). Auswirkung der Mindestmengenvereinbarung auf die stationäre Versorgungsstruktur. Das Gesundheitswesen.

[36] Wiesner R, Edwards E, Freeman R, Harper A, Kim R, Kamath P, Kremers W, Lake J, Howard T, Merion RM, Wolfe RA, Krom R (2003). Model for end-stage liver disease (MELD) and allocation of donor livers. Gastroenterology.

[37] Seider, H., Gaynor, M. and Vogt, W. B.: Volume-outcome and antitrust in US health care markets, Unpublished working paper (2004).

[38] Mesman R, Faber MJ, Berden BJJM, Westert GP (2017). Evaluation of minimum volume standards for surgery in the Netherlands (2003–2017): a successful policy?. Health Policy.

[39] Krautz C, Denz A, Weber GF, Gruetzmann R (2017). Influence of hospital volume effects and minimum caseload requirements on quality of Care in Pancreatic Surgery in Germany. Visceral Medicine.

